# The Effect of an Acute Bout of Moderate-Intensity Aerobic Exercise on Motor Learning of a Continuous Tracking Task

**DOI:** 10.1371/journal.pone.0150039

**Published:** 2016-02-22

**Authors:** Nicholas J. Snow, Cameron S. Mang, Marc Roig, Michelle N. McDonnell, Kristin L. Campbell, Lara A. Boyd

**Affiliations:** 1 Graduate Program in Rehabilitation Sciences, Faculty of Medicine, University of British Columbia, Vancouver, Canada; 2 School of Physical and Occupational Therapy, McGill University, Montréal, Canada; 3 Memory and Motor Rehabilitation Laboratory (MEMORY-LAB), Feil and Oberfeld Research Centre, Jewish Rehabilitation Hospital, Montréal Centre for Interdisciplinary Research in Rehabilitation (CRIR), Laval, QC, Canada; 4 International Centre for Allied Health Evidence and Alliance for Research in Exercise, Nutrition and Activity (ARENA), Sansom Institute for Health Research, School of Health Sciences, University of South Australia, Adelaide, Australia; 5 Graduate Program in Neuroscience, Faculty of Medicine, University of British Columbia, Vancouver, Canada; Eberhard Karls University of Tuebingen Medical School, GERMANY

## Abstract

**Introduction:**

There is evidence for beneficial effects of acute and long-term exercise interventions on several forms of memory, including procedural motor learning. In the present study we examined how performing a single bout of continuous moderate intensity aerobic exercise would impact motor skill acquisition and retention in young healthy adults, compared to a period of rest. We hypothesized that exercise would improve motor skill acquisition and retention, compared to motor practice alone.

**Materials and Methods:**

Sixteen healthy adults completed sessions of aerobic exercise or seated rest that were immediately followed by practice of a novel motor task (practice). Exercise consisted of 30 minutes of continuous cycling at 60% peak O_2_ uptake. Twenty-four hours after practice, we assessed motor learning with a no-exercise retention test (retention). We also quantified changes in offline motor memory consolidation, which occurred between practice and retention (offline). Tracking error was separated into indices of temporal precision and spatial accuracy.

**Results:**

There were no differences between conditions in the timing of movements during practice (*p* = 0.066), at retention (*p* = 0.761), or offline (*p* = 0.966). However, the exercise condition enabled participants to maintain spatial accuracy during practice (*p* = 0.477); whereas, following rest performance diminished (*p* = 0.050). There were no significant differences between conditions at retention (*p* = 0.532) or offline (*p* = 0.246).

**Discussion:**

An acute bout of moderate-intensity aerobic exercise facilitated the maintenance of motor performance during skill acquisition, but did not influence motor learning. Given past work showing that pairing high intensity exercise with skilled motor practice benefits learning, it seems plausible that intensity is a key modulator of the effects of acute aerobic exercise on changes in complex motor behavior. Further work is necessary to establish a dose-response relationship between aerobic exercise and motor learning.

## Introduction

The acquisition and retention of complex motor skills is crucial to the execution of most human motor behaviors, both throughout the lifespan as well as during recovery from neurological insult. [1] Converging evidence indicates that both single and repeated sessions of aerobic exercise are beneficial to both cognitive [2,3] and memory outcomes. [4,5] Recent work demonstrated that an acute aerobic exercise bout can facilitate the acquisition [6,7] and retention [6,8] of a complex motor skill, in young healthy adults, and enhance neuroplasticity in motor pathways believed to be implicated in skill learning. [6,9,10] However, existing evidence showing that pairing aerobic exercise with skilled practice can improve motor learning has, to date, favored acute bouts of high-intensity exercise. Firstly, Roig et al. [8] showed that performing 20 minutes of high-intensity cycling intervals at 90% peak power output (PO) facilitated the 24-hour and 7-day retention of a visuomotor accuracy-tracking task, compared to a resting control condition. Moreover, it was also found that exercise performed after motor practice had a greater benefit to long-term retention than exercise prior to practice. [8] More recently, Mang et al. [6] noted that 20 minutes of high-intensity cycling intervals (90% peak PO) performed before practicing a continuous tracking (CT) task [11] improved acquisition and 24-hour retention of the CT task, compared to a resting control condition. Specifically, participants showed significantly greater temporal precision in an implicitly learned sequence under the exercise condition. [6]

Studies highlight that the learning-oriented benefits of single and repeated bouts of aerobic exercise are both biological, affecting neuroendocrine processes, [6,9,12–14] and behavioral, manifesting through increases in cognitive processing, executive function, and attention. [2–5,15,16] Theoretically, acute bouts of high-intensity exercise stimulate the secretion of multiple neurochemicals that positively affect learning and neuroplasticity, and lead to enhanced motor memory consolidation. [13,17] For instance, Skriver et al. [13] found that elevated serum levels of blood lactate (BLa) and norepinephrine (NE) after high-intensity cycling intervals related to the magnitude of change associated with motor skill acquisition and retention, in a visuomotor accuracy-tracking task. Further, in the same study increased circulating brain-derived neurotrophic factor (BDNF) was related to the amount of motor skill change at retention testing. [13] Other work found relationships between post-exercise increases in catecholamines and verbal memory, [18] suggesting a potential link between transient increases in these circulating substances and memory processes.

There are numerous studies showing that acute and consistent participation in moderate-intensity aerobic exercise benefits aspects of cognitive [19] and executive functioning, [20] including attention and [15] reaction time [16]; stimulates the up-regulation of neurochemicals such as BDNF and NE [12,14]; and enhances neuroplasticity in the human motor system. [9,10] Moderate-intensity aerobic exercise has recently been shown to improve motor behavior. Specifically, 30 minutes of running at 65–85% age-predicted maximal heart rate (HR) was shown to improve the acquisition of a sequential visual isometric pinch task, compared to a control group; however, motor learning was not enhanced by the exercise bout. [7] There is potential for similar improvement in motor skill acquisition, or possibly motor learning, to carry over to distinctly different motor tasks, involving the recruitment of different neural resources. [21]

In the present study we examined how performing a single bout of continuous moderate-intensity aerobic exercise would impact the acquisition and retention of a motor skill in healthy adults. Participants practiced a CT task [6,11,22,23] after either 30 minutes of moderate-intensity cycling, or a rest period of equal duration, in a crossover fashion. During CT task practice we assessed motor skill acquisition. To assess motor learning we employed a delayed, no-exercise retention test, 24 hours after CT task practice. [24–26] We hypothesized that engaging in an acute bout of moderate-intensity cycling prior to performing the CT task would improve both the acquisition and retention of the complex motor skill, compared to rest.

## Materials and Methods

The present study was approved by the University of British Columbia (UBC)’s Clinical Research Ethics Board. All participants independently provided written and verbal informed consent, in accordance with the Declaration of Helsinki.

### Participants

Sixteen healthy adults (7 Females, 9 Males) were recruited from UBC and the surrounding community of Vancouver, British Columbia, Canada (see Results, Table 1). We included right-handed [27] volunteers who reported participating in ≥ 1500 metabolic equivalent of task [MET]-minutes•week^-1^ of physical activity, based on the long-form International Physical Activity Questionnaire (IPAQ). [28] Participants were also included if they were non-smokers, possessed an ability to read and understand English, and could maintain a seated, upright position for a prolonged period of time. Smokers were excluded on the basis that nicotine has been shown to influence memory performance. [29] Additional exclusion criteria included: a history of any neurological or psychiatric diagnoses (e.g., clinical depression); use of medication known to influence central nervous system activity; acute or chronic cardiorespiratory, musculoskeletal, or hormone-related (e.g., diabetes mellitus; eating disorders; obesity) disorders or conditions; a history of alcoholism or illicit drug dependency; visual or hearing impairment; acute or chronic contraindications to upper-extremity use; and contraindications to exercise (assessed via the Physical Activity Readiness Questionnaire [30]). Participants were also excluded if they drank an excess of six cups of coffee per day, [18] due to the possible effect of caffeine intake on memory performance. [31] Upon initial contact, participants received a written copy of the informed consent form, and were asked to self-report the above criteria.

**Table 1:** Participant Characteristics.

**Demographic**		**Mean (SD)**
	**Age (years)**	25.7 (3.1)
	**Height (cm)**	176.5 (9.4)
	**Body Mass (kg)**	69.3 (13.0)
**GXT**		**Mean (SD)**
	**VȮ**_**2peak**_ (mL•min^-1^•kg^-1^)	45.8 (7.10)
	**Peak PO** (Watts)	265.6 (41.3)
	**HR**_**peak**_ (beats•min^-1^)	184.8 (9.8)
	**RPE** (6–20)	18.1 (1.6)
	**[BLa]** (Mmol)	12.2 (3.0)
**Exercise Bout**		**Mean (SD)**
	**60% VȮ**_**2peak**_ (mL•min^-1^•kg^-1^)	27.5 (4.26)
	**PO** (Watts)	145.6 (32.0)
	**HR** (beats•min^-1^)	147.5 (12.7)
	**RPE** (6–20)	12.2 (1.4)
	**[BLa]** (Mmol)	4.8 (2.4)

Values presented as mean (SD). Age recorded in years; height recorded in cm; body mass recorded in kg. VȮ_2peak_, peak O_2_ uptake (mL•min^-1^•kg^-1^); PO, power output (Watts); HR, heart rate (beats•minute^-1^); RPE rating of perceived exertion (6–20 scale); [BLa], blood lactate concentration (Mmol); SD, standard deviation.

### Experimental Design

**Fig 1:**
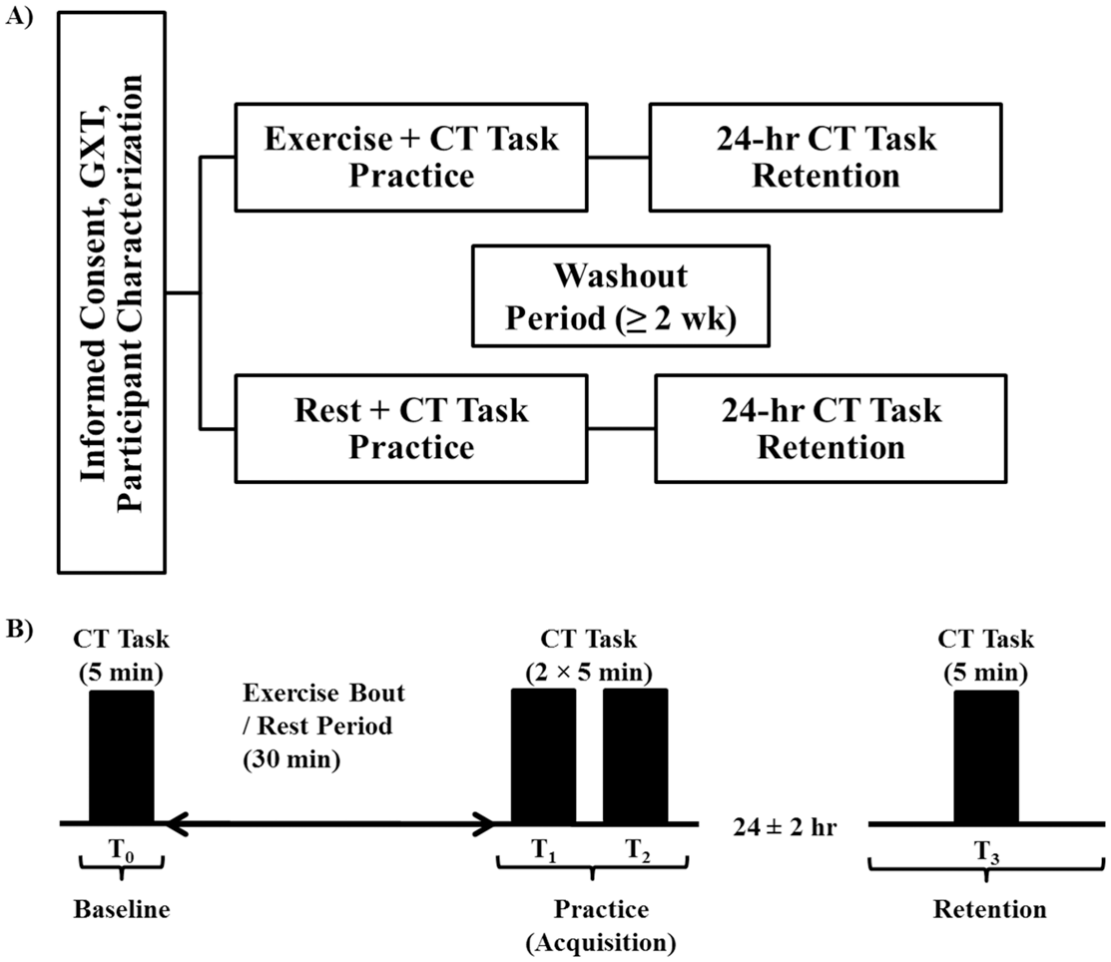
Diagrammatic representation of study design. The present study utilized a crossover design with repeated measures. **A)** Participants provided informed consent, underwent a graded exercise test (GXT) to exhaustion, and completed several screening and characterization questionnaires during the first experimental session. Participants were then pseudo-randomized to two experimental conditions including moderate-intensity aerobic exercise (based on GXT results) or seated rest prior to continuous tracking (CT) task practice. The CT task practice sessions were each followed by no-exercise retention test 24 ± 2 hours later. Experimental conditions were separated by a washout period of ≥ 2 weeks. **B)** During CT task practice sessions participants completed a single 5-minute tracking block (10 × 30-second trials) at baseline (T_0_). Thereafter, participants completed either 30 minutes of moderate-intensity cycling or seated rest, followed by two consecutive 5-minute tracking blocks at T_1_ and T_2_. Performance on practice blocks was used to index motor skill acquisition. Twenty-four ± 2 hours later, a 5-minute retention test was used to assess motor skill learning (T_3_).

The present study utilized a crossover design with repeated measures (Fig 1). During the initial experimental session all participants completed a graded exercise test (GXT) to exhaustion. Participants were then pseudo-randomized to complete one of two experimental conditions, prior to crossover: 1) moderate-intensity aerobic exercise; or 2) seated rest. The order of participation under each condition was counter-balanced across the study sample.

### Exercise Protocol

#### GXT

All participants completed a GXT, to determine their peak O_2_ uptake (V̇O_2peak_) for subsequent exercise prescription. Before attending this laboratory visit, participants were instructed to refrain from engaging in vigorous physical activity for ≥ 48 hours, ingesting alcohol for ≥ 6 hours, and eating for ≥ 2 hours. Upon arrival at the laboratory, participants completed several pre-screening questionnaires (see Participants), after which measurements of height and body mass were recorded in one layer of light clothes, with shoes removed. For the GXT, participants were outfitted with a silicone mouthpiece, a nose clip, and a one-way air valve (Hans Rudolph Inc., Shawkee, KS, USA). Participants’ HR was continually monitored via a Polar Wearlink^®^+ wireless HR transmitter and FS1 HR monitor watch (Polar Electro, Oy, Kempele, Finland). Throughout the GXT, measurements of V̇O_2_, CO_2_ output (V̇CO_2_), minute ventilation (V̇_E_), and respiratory exchange ratio (RER) were continuously monitored (5-second resolution) using a ParvoMedics TrueOne 2400 metabolic cart system (Sandy, UT, USA). The reliability and validity of this metabolic cart system have been established in previous research. [32] The GXT was completed on an electronically-braked Ergoline Ergoselect 200 cycle ergometer (Ergoline GmbH, Bitz, Germany). Briefly, exercise began at a PO of 50 Watts, for females, or 100 Watts for males—there was no formal warm-up period. For both females and males cycling resistance was incrementally increased by 30 Watts every 2 minutes, until the termination of the GXT. During cycling participants were instructed to maintain a pedaling cadence of 70–90 revolutions per minute (RPM). Participants had visual feedback of pedaling cadence, via a display mounted on the handlebars of the cycle ergometer. We also provided verbal feedback for the maintenance of cadence. At the end of every test stage (i.e., every 2 minutes), we recorded participants’ HR and rating of perceived exertion (RPE) using the Borg Scale (6–20 ratings). [33] Immediately after exercise cessation BLa concentration ([BLa]) was measured via finger-stick and an automated portable BLa analyzer and test strips (Lactate Pro, Arkray Inc., Kyoto, Japan); the validity of this device has been previously reported. [34] The GXT was terminated at volitional exhaustion, inability to maintain desired cadence or participant request to stop. Achievement of peak aerobic fitness was determined *post hoc* under the following conditions: HR > age-predicted maximal value, a plateau in V̇O_2_ and HR with further increases in workload, RER > 1.15, RPE > 17. [6,35,36] From the GXT, peak values of V̇O_2_, PO, HR, and RER were extracted (Table 1).

#### Standardized exercise bout

For 48 hours prior to each laboratory visit, participants were asked to refrain from vigorous exercise and alcohol consumption and were advised to get a normal night’s sleep. Each participant was tested at approximately the same time of day, to attenuate any diurnal fluctuations in motor memory processes. [37] Under the exercise condition, participants completed a 30 minute bout of cycling on a stationary cycle ergometer, at a PO corresponding to 60% V̇O_2peak_ (determined from the GXT) [38] and a pedaling cadence of 70–90 RPM. [6] Every 5 minutes HR and RPE were recorded. All participants were able to tolerate the originally prescribed exercise PO, with the exception of one individual. Because we aimed to examine effects of moderately intense exercise, PO was gradually reduced by 5 W increments from the initially prescribed intensity (110 W), until this participant’s RPE was within the “Moderate Intensity” range (11–14) [36]. The participant exercised at a PO of 80 W, and maintained an exercising HR and RPE of 126 beats•min^-1^ and 14, respectively. Upon completion of exercise, [BLa] was assessed using finger-stick. Under the exercise condition, this cycling bout immediately preceded CT task practice; whereas, under the resting condition CT task practice was preceded by 30 minutes of seated rest. Participants were asked to remain seated and relaxed for the entire rest period.

### CT Task

**Fig 2:**
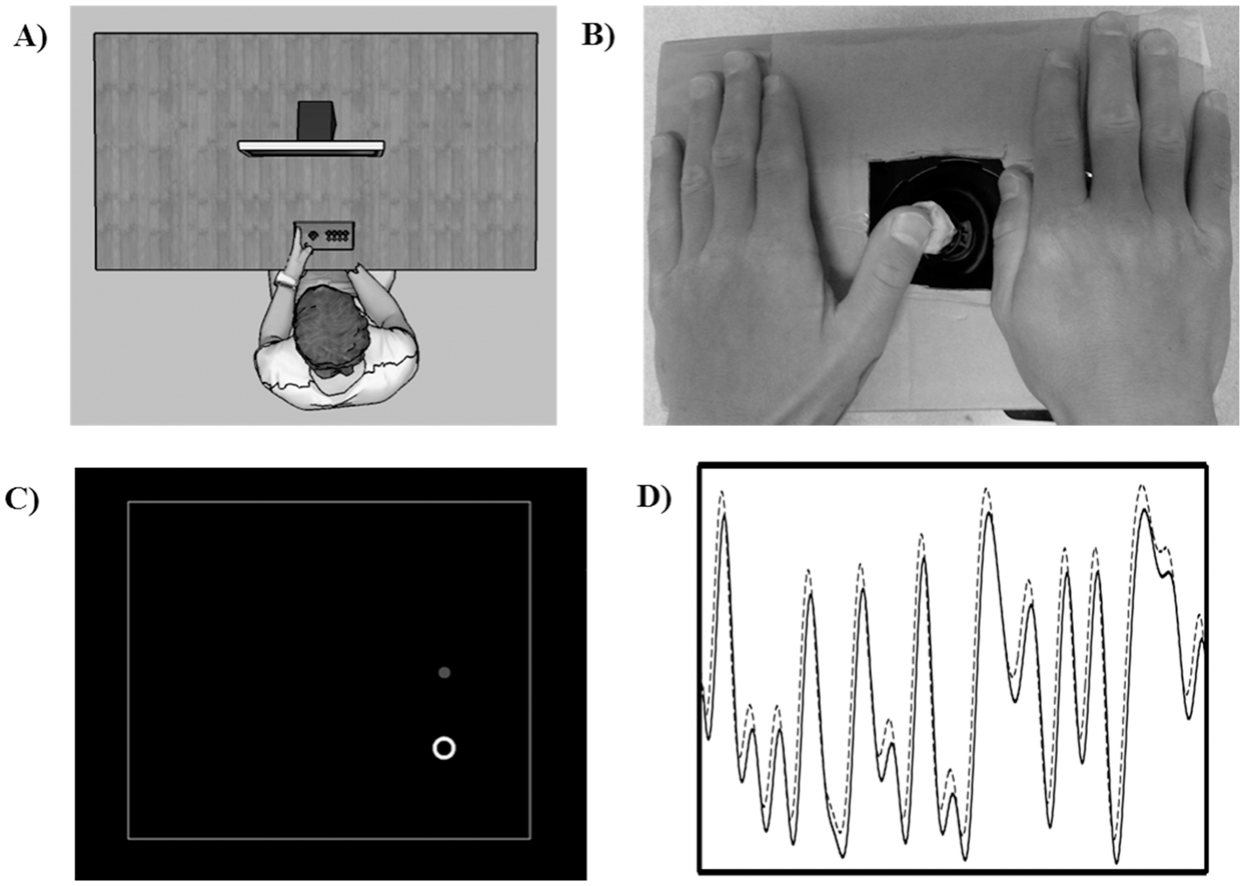
Schematic of the continuous tracking (CT) task used throughout study protocol. **A)** Participants were seated at a desk, in front of a computer monitor. **B)** A modified joystick was manipulated via abduction and adduction movements of the non-dominant hand. **C)** Participants’ view of the target (white ring) and cursor (red dot) presented on the computer monitor during CT task performance. **D)** A sample waveform used during a single CT task trial (30 seconds). The solid line represents a sample target sequence, whereas the dashed line depicts a participant’s movement trajectory during target tracking.

To examine the effect of a single bout of moderate-intensity aerobic exercise on motor skill acquisition and learning, participants practiced the CT task immediately after both exercise and rest conditions (Fig 1), followed 24 ± 2 hours later by a no-exercise retention test. Conditions were separated by a ≥ 2 week washout period, to prevent any order effect on subsequent practice. The CT task required the manipulation of a modified joystick (Logitech, Newark, CA, USA) via abduction and adduction movements of the non-dominant (i.e., left) thumb (Fig 2). All participants wore ear plugs and a noise-canceling headset during CT task practice and at the retention test.

The joystick was interfaced with a custom software program, developed using the LabVIEW platform (v. 9.0, National Instruments Corporation, Austin, TX, USA). [39] Joystick position sampling and all stimuli were presented at 50 Hz. Participants were seated in front of a computer monitor, and used joystick movements to control a cursor (a red dot), to track a moving target (a white ring which encircled the cursor) presented on a black background. Throughout tracking the target oscillated vertically, while moving right-to-left across the screen at a constant horizontal velocity.

The duration of a single trial (i.e., the amount of time it took the target to scroll across the screen) was 30 seconds. Each subsequent trial was preceded by a 2-second normalization period, during which the target (i.e., the white ring) and cursor (i.e., the red dot) were zeroed to their initial starting positions. One block of movements was made up of 10 × 30-second trials; participants completed: 1) one block at baseline, prior to the exercise bout or rest period (T_0_); 2) two blocks immediately after exercise or rest (T_1_ and T_2_); and 3) one block at the no-exercise retention session (T_3_). The purpose of the practice blocks T_1_ and T_2_ was to assess motor skill acquisition during early (T_1_) and late practice (T_2_), whereas the retention block (T_3_) examined motor skill learning. No rest was taken between acquisition blocks. Each trial was presented as a visual representation of a trigonometric series, constructed using the polynomial equation previously described by Wulf and Schmidt [40]:

$$f\left(x\right)\;=\;b_{0}+\;a_{1}sin\left(x\right)\;+\;b_{1}cos\left(x\right)\;+\;a_{2}sin\left(2x\right)\;+\;b_{2}cos\left(2x\right)\;+\;\ldots \;+\;a_{6}sin\left(6x\right)\;+\;b_{6}cos\left(6x\right).$$

These sequences were generated using coefficients (*a*, *b*) ranging between −10 and +10. [6] Coefficients were selected for each sequence such that the minimum and maximum positions of the sequence were of equal distance from the midline. [11] We have previously reported this method. [11] Each trial consisted of a movement sequence that was identical across participants and conditions, to ensure uniform difficulty. Difficulty was controlled for based on target movement range and velocity.

Prior to CT task practice we instructed participants to track the target with the cursor as accurately as possible at all times. For each participant, the direction of joystick control was reversed between exercise and rest conditions, such that left/right joystick movements corresponded to up/down cursor movements for one condition and down/up cursor movements for the other. Additionally, the order of sequence presentation (i.e., regular presentation, reversed presentation) was reversed between conditions. By reversing both joystick directionality and sequence presentation between conditions, movements were identical across conditions. Participants were explicitly informed only of the direction of joystick control at the beginning of each session. Movement directionality was the same for practice and retention sessions under each condition; and directionality across conditions was pseudo-randomized and counterbalanced across the sample. We controlled for movement difficulty and directionality, in order to be able to quantify potential learning across trial repetitions and conditions. Participants were not provided error feedback during or after tracking practice.

### Data Analyses

All CT task data were processed using a custom MATLAB script (Version R2013b, The Mathworks, Inc., Natick, MA, USA). Data from each individual trial were collapsed to provide a measure of tracking performance within each block, and to make comparisons across tracking blocks.

Participants’ motor performance was evaluated based on changes in spatial accuracy and temporal precision. To accomplish this, participants’ absolute root-mean-square error (RMSE) [41] of tracking was separated into temporal and spatial components using a time series analysis (TSA). [11,22] In the TSA, participants’ tracking patterns from each trial are cross-correlated with the target pattern until a maximum correlation coefficient (R^2^) is reached. The cross-correlation coefficients reflect the spatial accuracy of participants’ tracking performance, while the distance (number of samples, multiplied by 5 milliseconds) that tracking data are shifted along the target data sequence to achieve the maximum R^2^ represents participants’ temporal precision. Spatial accuracy is reported as shifted RMSE and temporal precision is reported as time lag. Lower shifted RMSE score indicates greater spatial tracking performance. Time lag scores in larger negative numbers indicate greater time lag of tracking, while a zero value represents no tracking time lag between participant movements and the target; any trial including a positive time lag value was omitted. Thus, measures of temporal precision (time lag) and spatial accuracy (shifted RMSE) were calculated separately, to evaluate tracking error across practice and at retention. [11] Tracking performance was decomposed into temporal and spatial dimensions because these aspects of procedural memory have been shown to evolve distinctly from one another, [42] involve separate neural pathways, [11,42] and have been shown to be differentially impacted by an acute bout of aerobic exercise. [6] To account for possible differences in tracking performance at baseline (T_0_), all data from acquisition (T_1_, T_2_), and retention (T_3_) were analyzed as a change score from T_0_. Additionally, a change score was calculated between performance at T_2_ and T_3_, to assess offline motor memory consolidation. [23,24] More negative time lag change scores indicate greater temporal precision, whereas greater positive shifted RMSE change scores reflect greater spatial accuracy.

### Statistical Analyses

Data distributions and assumptions were tested using the Shapiro-Wilk test and visual inspection of histogram plots. Omnibus statistical tests were conducted via repeated-measures analyses of variance (rmANOVAs) and paired-samples *t*-tests.

Motor skill acquisition was characterized by performance changes that occurred during motor practice, (26) and was assessed using separate two-way Condition (exercise vs. rest) by Time (T_0_-T_1_ vs. T_0_-T_2_) rmANOVAs with change score values of time lag and shifted RMSE as the dependent variables. In the event of a significant Condition × Time interaction effect post hoc pairwise comparisons were made separately for each condition across levels of time, using the Bonferroni correction.

Motor learning was assessed using the 24-hour no-exercise delayed retention test, [26] and was evaluated across conditions via separate paired-samples *t*-tests on time lag and shifted RMSE change scores (T_0_-T_2_ change scores). Additionally, offline motor memory consolidation was tested using paired-samples *t*-tests on participants’ change-score in time lag and shifted RMSE, calculated between T_2_ and T_3_. [7,23,24] Due to theoretical distinctness between the above concepts of motor skill acquisition, learning, and consolidation, [24,26] separate statistical tests were used for each. Similar statistical approaches have been previously employed. [7,23] Statistical significance was set at *p* ≤ 0.05. Results are reported as mean ± standard error of mean (SEM), unless otherwise indicated. Effect sizes are reported as Cohen’s *d* [43], and were calculated as ([M_1_ –M_2_]/[σ_pooled_]), where [M_1_ –M_2_] is the mean difference between two measurements and [σ_pooled_] is the pooled standard deviation of those two means. Statistical tests were performed using SPSS (V23.0, IBM Corporation, Armonk, New York, USA). Effect sizes were calculated using Microsoft Excel 2010 (Redmond, WA, USA).

## Results

### Participants

Of the 16 participants, nine were male and seven were female, with an overall mean age of 25.7 (0.8) years (Table 1). Participants reported an average of 4136.3 (413.2) MET-minutes•week^-1^ of moderate- to-vigorous leisure time physical activity; and the mean V̇O_2peak_ for males was 47.5 (2.3) mL•min^-1^•kg^-1^ and 43.6 (2.7) mL•min^-1^•kg^-1^ for females, corresponding to “excellent” fitness for both males and females. [36] The mean PO, HR, RPE and post-exercise [BLa] readings for the continuous exercise bout were 167 (7) Watts, 151 (4) beats•minute^-1^, 12 (1), and 6.4 (0.7) Mmol for males; and 119 (9) Watts, 143 (5) beats•minute^-1^, 12 (0), and 2.8 (0.3) Mmol for females, respectively.

### Data Inspection

All CT task data were deemed normally distributed on the basis of non-significant Shapiro-Wilk statistics (*W*_(16)_ = 0.900–0.977, *p* = 0.081–0.934), as well as upon visual inspection of histogram plots.

### Temporal Precision (Time Lag of Tracking)

**Fig 3:**
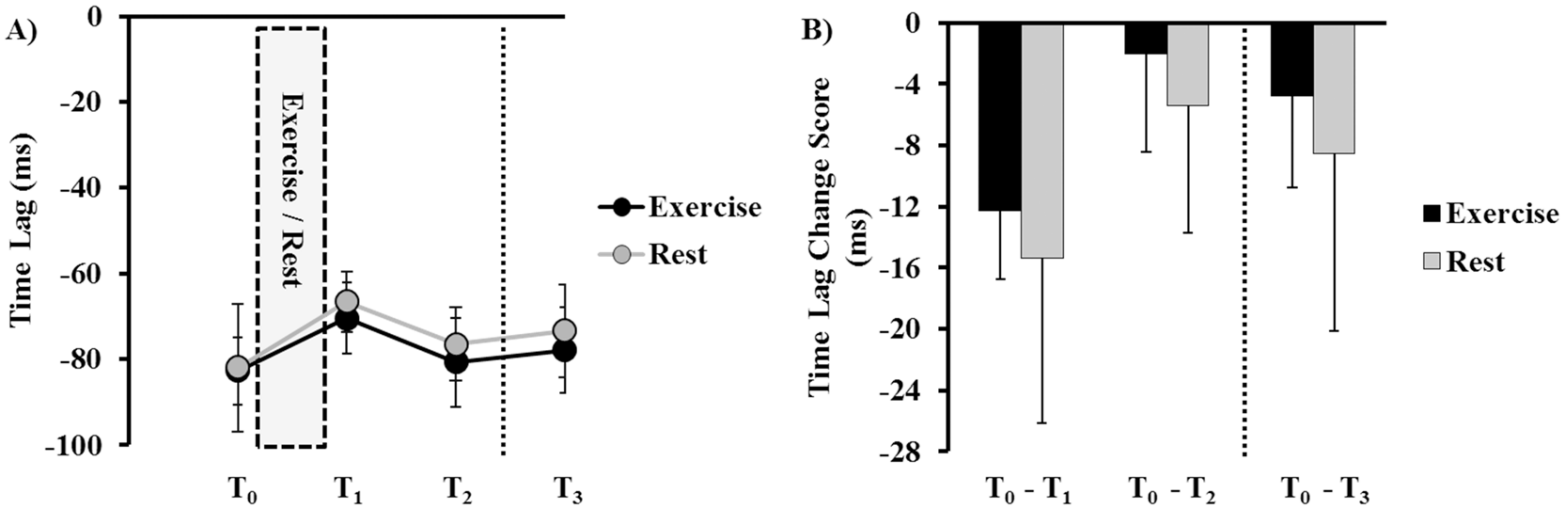
Temporal precision (time lag) performance on the continuous tracking (CT) task. **A)** Raw time lag values at baseline (T_0_), acquisition (T_1_, T_2_), and retention (T_3_) under exercise (black line) and rest (grey line) conditions. Less negative time lag values indicate greater temporal precision. The inlaid box represents the 30-minute exercise bout or rest period. **B)** Time lag change scores between baseline, acquisition (T_0_-T_1_, T_0_-T_2_), and retention (T_0-_T_3_) blocks, under exercise (black bars) and rest (grey bars) conditions. More negative change scores indicate greater temporal precision. There was no significant difference between conditions during acquisition and retention measurements (*p* > 0.05). The vertical dotted lines in **A** and **B** represent the 24 ± 2 hours between CT practice and retention days. Error bars in **A** and **B** represent mean ± standard error of mean (SEM).

Group plots of time lag by time-point (T_0_, T_1_, T_2_, T_3_), under the exercise and rest conditions, are depicted in Fig 3A. Group plots of time lag change score by time-point (T_0_-T_1_, T_0_-T_2_, T_0_-T_3_) are illustrated in Fig 3B.

The two-way rmANOVA on change score values of time lag during skill acquisition (T_0_-T_1_, T_0_-T_2_) demonstrated a trend towards a significant main effect of Time (*F*_(1, 15)_ = 3.919, *p* = 0.066, *d* = 0.47). Otherwise, there was neither a significant main effect of Condition (*F*_(1, 15)_ = 0.101, *p* = 0.756, *d* = 0.11), nor a significant Condition × Time interaction (*F*_(1, 15)_ = 0.003, *p* = 0.956 *d* = 0.19).

The paired-samples *t*-test on retention change scores (T_0_-T_3_) highlighted that there was no effect of Condition (*t*_(15)_ = 0.310, *p* = 0.761, *d* = 0.11). There was no difference in temporal precision between exercise and rest conditions at retention.

In terms of offline motor memory consolidation (T_2_-T_3_ change score), the paired-samples *t*-test demonstrated no effect of Condition (*t*_(15)_ = 0.043, *p* = 0.966, *d* = 0.01). Thus, offline consolidation of time lag of tracking for the CT task did not differ between exercise and rest conditions.

### Spatial Accuracy (Shifted RMSE)

**Fig 4:**
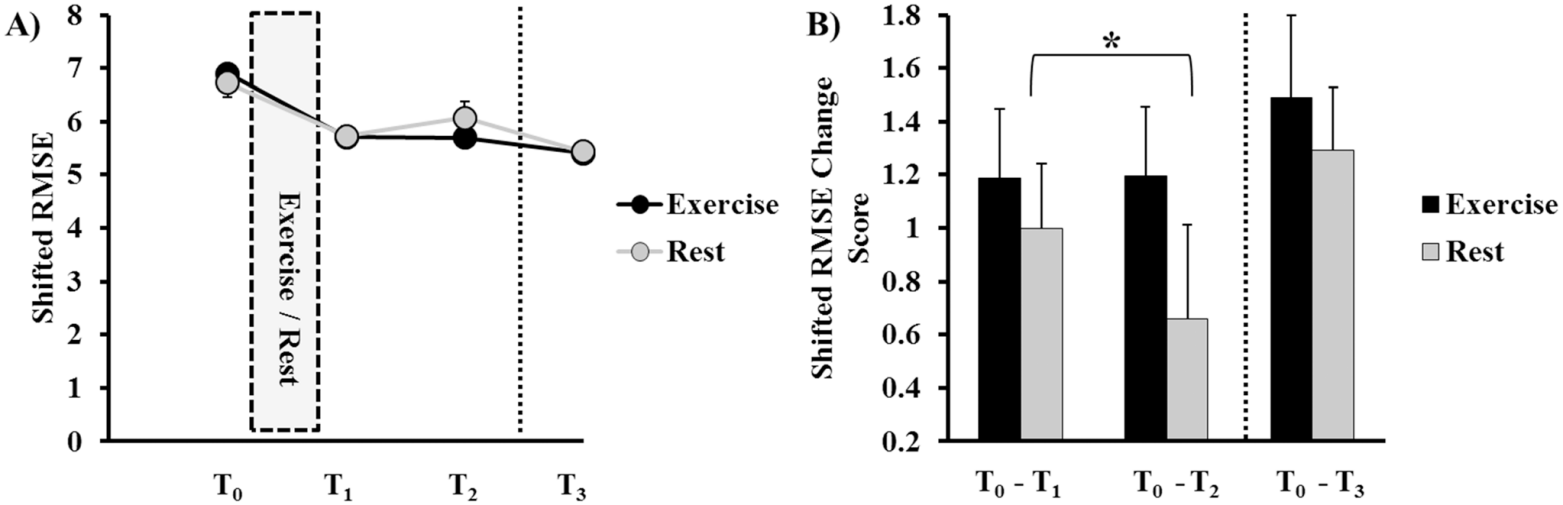
Spatial accuracy (shifted root-mean-square error [RMSE]) performance on the continuous tracking (CT) task. **A)** Raw shifted RMSE values at baseline (T_0_), acquisition (T_1_, T_2_), and retention (T_3_) under exercise (black line) and rest (grey line) conditions. Smaller shifted RMSE values indicate greater spatial accuracy. The inlaid box represents the 30-minute exercise bout or rest period. **B)** Shifted RMSE change scores between baseline, acquisition (T_0_-T_1_, T_0_-T_2_), and retention (T_0-_T_3_) blocks, under exercise (black bars) and rest (grey bars) conditions. Greater change scores indicate greater spatial accuracy. Additionally, performance was significantly reduced from the first to the second acquisition block under the rest condition (*p* = 0.05). Spatial accuracy did not differ between conditions at retention (*p* > 0.05). The vertical dotted lines in **A** and **B** represent the 24 ± 2 hours between CT practice and retention days. Error bars in **A** and **B** represent mean ± standard error of mean (SEM).

Group plots of shifted RMSE by time-point (T_0_, T_1_, T_2_, T_3_), under the exercise and rest conditions, are shown in Fig 4A. Group plots of time lag change score by time-point (T_0_-T_1_, T_0_-T_2_, T_0_-T_3_) are displayed in Fig 4B.

The two-way rmANOVA on change scores (T_0_-T_1_, T_0_-T_2_) during motor skill acquisition showed no significant main effects of Condition (*F*_(1, 15)_ = 1.292, *p* = 0.274, *d* = 0.34) or Time (*F*_(1, 15)_ = 0.916, *p* = 0.354, *d* = 0.18). However, the rmANOVA revealed a significant Condition × Time interaction effect (*F*_(1, 15)_ = 4.396, *p* = 0.050, *d* = 0.66). Pairwise comparisons showed that, under the rest condition, spatial accuracy worsened from T_1_ to T_2_ (*t*_(15)_ = 1.680, *p* = 0.050, *d* = 0.29), but performance was stable from T_1_ to T_2_ under the exercise condition (*t*_(15)_ = -0.059, *p* = 0.477, *d* = 0.01). These results indicate that participants were able to maintain tracking performance for a longer time, under the exercise condition; whereas under the rest condition, there was a decay in the spatial aspect of tracking performance.

At retention (T_0_-T_3_ change score), the paired-samples *t*-test indicated that there was no difference in spatial accuracy (*t*_(15)_ = 0.640, *p* = 0.532, *d* = 0.19) between exercise and rest conditions.

The paired-samples *t*-test on offline consolidation change scores (T_2_-T_3_) showed that participants’ motor memory consolidation of spatial performance did not differ between exercise and rest conditions (*t*_(15)_ = 1.208, *p* = 0.246, *d* = 0.37). Thus, there was no difference in motor learning or offline motor memory consolidation between conditions.

## Discussion

The primary aim of the present study was to determine the effect of a single 30-minute bout of moderate-intensity cycling (PO corresponding to 60% V̇O_2peak_) on the acquisition and retention of a complex motor skill (CT task), in a sample of healthy young adults. We hypothesized that exercising at a moderate intensity before practicing the CT task would lead to significantly improved motor skill acquisition and retention, compared to a rest period of equal duration. We discovered that, compared to rest, exercise appeared to facilitate the maintenance of motor performance throughout the acquisition phase; however, contrary to our primary hypothesis, we found that moderate-intensity exercise did not influence indices of motor skill learning, nor did it affect offline motor memory consolidation.

It has become increasingly evident that there is a complex interaction between exercise intensity and motor memory. [24–26] Several recent works have examined the role of acute aerobic exercise in modulating the acquisition and retention of complex motor skills. [6–8] The first of these studies showed that performing high-intensity intermittent aerobic exercise in close temporal proximity to motor skill practice enhanced measures indicative of both motor skill acquisition (Mang et al. [6]) and retention (Roig et al. [8], Mang et al. [6]). More recently, Statton et al. [7], demonstrated that a single bout of moderate-intensity running enhanced motor skill acquisition in a motor task distinct from that utilized by our group and other existing work. However, in the aforementioned study, effects of acute exercise on motor learning were not examined as there was no delayed retention test following the exercise bout. [7] Other work has suggested that moderate-intensity exercise may have protective effects against motor memory interference, [44] or serve to enhance motor cortical excitability in response to skilled motor practice. [45] The present findings add to our understanding of how acute exercise affects skill acquisition, showing that when delivered within a single session moderate-intensity efforts have little effect on changes in performance associated with motor learning.

Taken with prior evidence, our results suggest that exercise effects on motor behavior are not universal, and may be task-dependent, [21] and/or reliant on the outcome measure used to assess motor performance. [7] With the present task and participant characteristics, we believe that exercising at a high-intensity may be necessary to drive lasting changes in motor behavior, when delivered in close proximity to skilled motor practice. When motor practice and moderate-intensity exercise are paired over multiple sessions, there appears to be an additive effect on motor skill acquisition. [7] The use of acute and long-term interventions in combination could maximize the effects that cardiovascular exercise has on human procedural memory. [4]

The observed effect of moderate-intensity exercise on online performance, shown here and elsewhere, [7] agrees with previous literature examining the cognitive and neural effects of acute moderate-intensity aerobic exercise. [2] Although the motor task employed in the present work is not a central executive task, and involves the recruitment brain areas distinct from tests involved in these paradigms, [42] exercise-induced changes in executive function are expected to impact performance during motor skill acquisition. [4] Meta-analyses have concluded that acute and long-term participation in moderate-intensity exercise can enhance executive function, [2,3] working memory, [5] and short- and long-term (non-motor) memory, when provided in conjunction with behavioral tasks. [4] Such work supports the idea that stabilized motor performance after moderate-intensity aerobic exercise could be related, in part, to exercise-induced enhancements in cognitive processes and underlying neural correlates. [46] It should also be noted that moderate-intensity aerobic exercise has been shown to promote motor cortical plasticity, as assessed using transcranial magnetic stimulation, [10] and increases intracortical excitability in non-exercised upper-limb motor cortical representations. [47,48] It has been thus suggested that moderate-intensity exercise promotes a favorable cortical environment for learning-induced plasticity, [45,49] but that exercise intensity may need to be greater in order to translate to lasting changes in motor cortical excitability and improved motor learning [45,46,50]

While we demonstrated a *relative* improvement in motor skill acquisition after exercise, compared to rest, we found no differences in motor skill retention or offline consolidation scores, indicating no apparent lasting effect on motor learning. It is possible that these observations are related to differences in the neurochemical consequences of moderate- versus high-intensity aerobic exercise protocols. Evidence indicates that high-intensity exercise influences on memory are correlated with increased circulating levels of catecholamines, growth factors, and of other substrates. [13,14,18,51] There is evidence that increases in the concentration of peripheral neurochemicals (e.g., BNDF, BLa, catecholamines) after a single bout of high-intensity aerobic exercise are associated with improvements in different types of memory, [13,18] including that of performances related to motor learning. [13] However, as evidenced by our previous work, this finding is not universal. [6] Importantly, several of these substances (e.g., BDNF, catecholamines) have been shown to be necessary for the formation of memories. [52] The evidence in relation to moderate-intensity, in contrast, is less consistent; work from elsewhere indicates that exercise-induced up-regulation of these memory-enhancing substances may be intensity-dependent. [13,18] Although, recent studies demonstrate that an acute bout of exercise performed at a moderate-intensity improves declarative memory, [12,53] and these improvements are associated with exercise-induced increases in neuro-adrenergic activation. [12]

Another more practical consideration regarding exercise effects on motor learning entails the timing of the exercise bout relative to memory trace exposure and memory testing. [4,8,26,53] Previous work examining the effects of 30 minutes of moderate-intensity cycling (RPE 13–15) on oral paragraph recall suggests that timing the exercise bout prior to the memory trace exposure has a greater effect on memory than exercising after exposure. [53] Conversely, high-intensity exercise performed after skilled motor practice enhances its effect on long-term retention (1 week), with no effect on 1-hour retention, and no benefit on 1-day retention over exercising prior to motor practice. [8] Another study found that 6 minutes of moderate-intensity cycling (70% V̇O_2peak_) after exposure to emotionally arousing images enhanced recognition in healthy elderly individuals and elderly persons with mild cognitive impairment, as determined using a surprise 1 hour post-exercise memory test. [12]

It is possible that the above declarative memory tasks could be more susceptible to improvements with exercise, due to the higher emotional content of the information to be remembered. This is consistent with studies showing that endogenous stress hormones released with exercise modulate memory for experiences that induce their release, and that the degree of arousal at encoding modulates memory improvement. [54] Another important difference concerns the placement of the retention test. Previous work using memory tests performed 35 minutes and 1 hour after exposure allowed researchers to observe short-term improvements in memory, [12,53] whereas in our study, we performed the retention test 24 hours after practice. Perhaps the intensity used in this experiment, compared to our previous findings, [6] was not enough to elicit improvements in long-term memory observable 24 hours after exposure. Although it has been suggested that a within-session delayed memory test can increase the available time to support short-term memory consolidation, [53] this timing effect depends on several factors independent of exercise intensity including the age and physical fitness of participants, [55,56] the form of the memory being examined (i.e., declarative vs. procedural, emotional vs. neutral), [57] and the nature of the memory test (i.e., explicit vs. implicit, free recall vs. recognition). [24,57] Nevertheless, we cannot discount the possibility that a short-term delayed retention test (30–60 minutes post-exercise) may have revealed positive exercise effects not observed under the current conditions.

In the present study we found that 30 minutes of cycling at a PO corresponding with 60% VȮ_2peak_ resulted in improved motor memory encoding at the end of the acquisition period, relative to a rest period of equivalent duration. Specifically, improved encoding came as a result of maintained motor skill performance after exercise, while performance decreased over time after rest. In the current work we utilized two blocks of CT task practice, consisting of a total of 20, 30-second trials. Albeit a similar task was used by Mang et al. [6], with an equivalent dose of practice, other work from our laboratory has prescribed a much larger practice dose, in terms of block duration, number of blocks, and number of practice days. [11,22,23] While we found that acute moderate-intensity aerobic exercise was insufficient to improve learning of the CT task, despite an improvement in both performance and change associated with learning after high-intensity exercise, [6] it is possible that with more sustained practice after moderate-intensity exercise could have a beneficial effect on motor learning. Here, we consider a low practice dose a potential limitation of the present study. Previous literature has described improvements in motor skill acquisition after a long-term exercise intervention in the absence of continued motor practice. [58,59] Furthermore, there is now evidence that pairing motor practice and moderate-intensity exercise over multiple sessions can promote improvements in motor behavior, during skill acquisition. [7] It is possible that we may have seen similar results in the presence of a larger acute practice dose, or multiple practice sessions.

## Conclusions

We showed that a single bout of moderate-intensity aerobic exercise has the ability to modulate motor skill performance *relative* to a period of rest, but in isolation did not affect motor skill learning. In order to design and explore novel interventions that can augment existing rehabilitation practice, we must elucidate the appropriate dose-response relationship (i.e., intensity, duration, mode, and frequency), between aerobic exercise and motor learning.
